# Compositional Characteristics and In Vitro Evaluations of Antioxidant and Neuroprotective Properties of Crude Extracts of Fucoidan Prepared from Compressional Puffing-Pretreated *Sargassum crassifolium*

**DOI:** 10.3390/md15060183

**Published:** 2017-06-18

**Authors:** Wen-Ning Yang, Po-Wei Chen, Chun-Yung Huang

**Affiliations:** Department of Seafood Science, National Kaohsiung Marine University, No. 142, Haijhuan Rd., Nanzih District, Kaohsiung 81157, Taiwan; amy0933206716@gmail.com (W.-N.Y.); a3989889@gmail.com (P.-W.C.)

**Keywords:** antioxidant, fucoidan, neuroprotection, rat pheochromocytoma PC-12 cells, *Sargassum crassifolium*

## Abstract

Fucoidan, a fucose-containing sulfated polysaccharide with diverse biological functions, is mainly recovered from brown algae. In this study, we utilized a compressional-puffing process (CPP) to pretreat *Sargassum crassifolium* (SC) and extracted fucoidans from SC by warm water. Three fucoidan extracts (SC1: puffing at 0 kg/cm^2^; SC2: puffing at 1.7 kg/cm^2^; and SC3: puffing at 6.3 kg/cm^2^) were obtained, and their composition, and antioxidant and neuroprotective activities were examined. The results suggest that CPP decreased the bulk density of algal samples, expanded the algal cellular structures, and eliminated the unpleasant algal odor. The extraction yields of fucoidans were increased and impurities of fucoidans were decreased by increasing the pressures used in CPP. The SC1–SC3 extracts displayed various characteristics of fucoidan as illustrated by the analyses of composition, Fourier transform infrared (FTIR) spectroscopy, and molecular weight. All three extracts SC1–SC3 showed antioxidant activity dose-dependently. Although both SC1 and SC2 possessed high and similar neuronal protective properties, SC2 showed a higher extraction yield, higher efficacy in the reversion of H_2_O_2_-induced cytotoxicity in rat pheochromocytoma PC-12 cells, and lower impurities compared with SC1, and thus SC2 is suggested as a good candidate for a therapeutic agent in the preventive treatment of neurodegenerative diseases.

## 1. Introduction

Reactive oxygen species (ROS), which include hydroxyl radical, superoxide anion, hydrogen peroxide (H_2_O_2_), and singlet oxygen, are usually produced and degraded by all aerobic organisms, leading to either physiological concentrations required for normal cell function, or excessive ROS quantities, resulting in a phenomenon termed oxidative stress [1]. Excessive exposure to oxidative stress may damage cellular DNA, proteins, and lipids; alter biochemical compounds; and corrode cell membrane. Therefore, ROS play a critical role in the development of various diseases such as cancer, atherosclerosis, respiratory ailments, and even neuronal death [2].

As the population ages, the prevalence of neurodegenerative diseases such as Alzheimer’s disease (AD) and Parkinson’s disease (PD) is increasing. The brain and nervous systems are known to be prone to oxidative damage. Multiple lines of evidence have demonstrated that oxidative stress-induced cell damage is involved both in the physiological process of aging and in neurodegenerative diseases such as AD and PD [3,4]. Thus, it may be possible to develop a therapeutic strategy by augmenting or fortifying endogenous defense against oxidative stress through dietary or pharmacological intake of antioxidants. Hence, there is an urgent need to identify natural substances that can scavenge free radicals and protect neuronal cells from oxidative damage, without notable side effects.

Marine algae are recognized as rich sources of biologically active compounds with great pharmaceutical and biomedical potentials, which include the capacity to exert anticoagulant, anti-viral, antioxidant, anti-allergic, anti-cancer, anti-bacterial, anti-diabetic [5], and anti-inflammatory [6] effects. Scientific studies have also provided insight into neuroprotective properties of marine algae [7]. Reports demonstrated that *Sargassum thunbergii* extract may be an effective therapeutic and preventative herbal extract for the treatment of several neurodegenerative and oxidative stress-related diseases [8]. Moreover, heteropolysaccharides were extracted from *Sargassum integerrimum* (SI), *Sargassum maclurei* (SM), *Sargassum naozhouense* (SN), *Spiraea thunbergii* (ST), *Sargassum hemiphyllum* (SH), and *Sargassum fusiforme* (SF) and their neuroprotective effects were evaluated. The investigators found that SI, SN, ST, and SF exhibited neuroprotective activities, whereas SH and SM did not [9]. These results clearly demonstrated that the polysaccharide extracts from *Sargassum* spp. may actually provide a neuroprotective effect; however, the efficacy of these extracts may also depend on a variety of factors such as marine algal species, extraction methods, and the physicochemical characteristics of extracts. Thus, a thorough analysis of polysaccharide extracts from *Sargassum* spp. and their neuroprotective effects is warranted.

Fucoidan comprises a group of fucose-rich sulfated polysaccharides containing varying amounts of galactose, mannose, xylose, and glucuronic acid. It can be extracted from marine brown algae such as *Sargassum* spp. and *Fucus* spp. [10]. Fucoidan has been widely documented to exhibit biological activities including antitumor and immunomodulatory, antivirus, antithrombotic and anticoagulant, anti-inflammatory, and effects against various renal, hepatic and uropathic disorders [11]. However, the neuroprotective effects of fucoidan are not well understood. A previous study has demonstrated that a commercially available fucoidan with a high purity (more than 98.0%) can protect neurocytes against H_2_O_2_-induced apoptosis by reducing ROS levels and activating the PI3K/Akt signaling pathway [12]. However, obtaining this high-purity fucoidan requires complicated extraction and purification processes and a high cost of production, which limit its commercial applications. Therefore, it is crucial to examine the neuroprotective effects of alternative agents such as the crude extracts of fucoidan.

The present study builds upon on the research reported in our previous investigation [13]. In brief, an oven-dried brown seaweed *Sargassum crassifolium* (SC) harvested from the southern coastal area of Taiwan was subjected to compressional-puffing at different pressures and then fucoidan was extracted using warm water. The recovered crude extracts of fucoidan were evaluated with respect to their extraction yields, composition, and biological functions including antioxidant and neuroprotective activities. To the best of the authors’ admittedly limited knowledge, this is the first report to evaluate the neuroprotective effects of crude extracts of fucoidan obtained from compressional-puffing-pretreated SC. In addition, we aimed to demonstrate the reversal of H_2_O_2_-induced neuronal cellular apoptotic indices using crude extracts of fucoidan and explore its potential as a natural neuroprotective agent.

## 2. Results and Discussion

### 2.1. Effects of Compressional-Puffing Parameters on the Characteristics of Puffed Algal Samples

**Figure 1:**
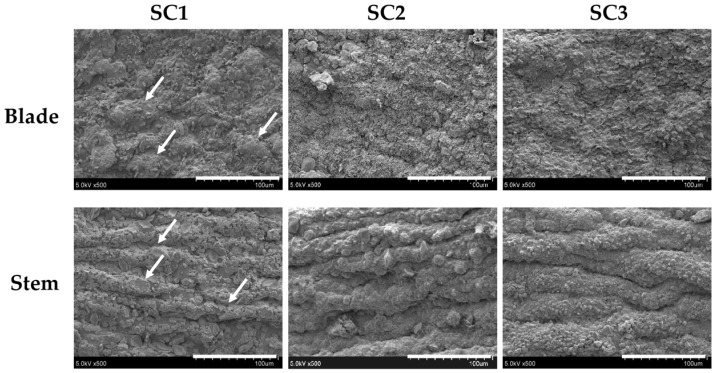
SEM photographs of blade and stem cells from SC for SC1, SC2, and SC3. The intact blade and stem cellular structures are indicated by white arrows. The scale bar (100 μm) is also shown in each graph.

The sample of SC used in this study was composed of 2.36% protein, 0.98% lipid, 33.98% ash, and 62.67% carbohydrate (dry basis). A comparison of various *Sargassum* spp., namely, *S. cristaefolium*, *S. horneri*, and *S. glaucescens*, revealed that *S. cristaefolium* possessed the highest carbohydrate content (69.87%), followed by SC (62.67%), *S. horneri* (61.82%), and *S. glaucescens* (59.52%) [13,14]. These data indicate that SC also possesses a relatively high amount of carbohydrate, which is suitable for extraction of fucoidan. Before extraction of fucoidan, the algal sample was pretreated with a compressional-puffing process (CPP). The CPP has advantages for increasing the extraction yields of fucoidan from brown seaweed [13] and augmenting the extraction yields of total phenolics and total flavonoids from pine needles [15]. The operational parameters for CPP were set as follows: mechanical compression pressure (5 kg/cm^2^), number of compression times (3), puffing temperatures (0, 140, and 180 °C), and reaction time (10 s) (Table 1). Subsequently, the powder of SC (weight = 2.5 g, H_2_O = 8.1%) was automatically fed into the chamber and the corresponding puffing pressures inside the chamber were 1.7 and 6.3 kg/cm^2^ for temperatures 140 and 180 °C, respectively (Table 1). The degree of moisture loss in puffed SC was indicated. When the pressure reached 1.7 kg/cm^2^, 14.11% ± 2.36% moisture loss for SC2 occurred. When the pressure was increased to 6.3 kg/cm^2^, the moisture loss for SC3 was 34.69% ± 6.02% (Table 1). Therefore, the degree of moisture loss in puffed algal sample was increased by raising the puffing pressures. A previous report showed that increased puffing pressure resulted in greater extent of browning in puffed samples [16]. To evaluate the color difference between non-puffed and compressional-puffed algal samples, the Hunterlab *L*, *a*, and *b* values and blackness of SC1–SC3 were measured. Data presented in Table 1 show that the *L*, *a*, and *b* values varied significantly among SC1–SC3 (*p* < 0.05) and blackness rose significantly (*p* < 0.05) as puffing pressure increased, indicating that the heat and pressure in CPP may cause the brown color of puffed algal samples to become darker. Expansion of volume is a puffing behavior that varies under different puffing conditions, such as temperature, processing time, and pressure [17]. The volume expansion of compressional-puffed algal samples can be characterized by analyzing the bulk density. The bulk densities for SC1, SC2, and SC3 were 0.54 ± 0.00, 0.52 ± 0.00, and 0.50 ± 0.00 g/mL, respectively, which suggests that CPP may expand the volume of algal samples and simultaneously decrease their bulk density (Table 1). The occurrence of expansion in compressional-puffed algal samples can also be directly observed by scanning electron microscopy (SEM). Figure 1 shows the result of microscopic examinations of tissues (blade and stem) of SC for SC1, SC2, and SC3. It was found that SC1 (non-puffed) showed intact blade and stem cellular structures (indicated by white arrows). In contrast, the blade and stem cellular structures in SC2 (puffed at pressure = 1.7 kg/cm^2^) and SC3 (puffed at pressure = 6.3 kg/cm^2^) were expanded, lost regular cellular compartments, and exhibited a disrupted arrangement. There are a variety of volatile compounds in marine algae that give seaweed its characteristic odor [18]. The seaweed smell of marine algae is sometimes considered unpleasant and pungent, which has limited its use in commercial food and cosmetic products. A number of methods exist that are capable of removing the unpleasant odor from marine products, including a combined method of solvent extraction and spray drying [19], as well as techniques which involve the use of activated carbon, ion exchange resin, and calcium carbonate [20]. However, these methods are time- and reagent-consuming, and complicated. In the present study, CPP was found to be effective at eliminating the unpleasant seaweed smell in a marine product. We performed sensory evaluation analysis of algal samples before and after CPP using a seven-point hedonic scale. The results presented in Table 1 suggest that SC3 had the highest score, representing the highest degree of liking for odor (4.60 ± 0.84), followed by SC2 (3.70 ± 0.97), and then SC1 (2.67 ± 0.79), indicating that the elevated pressures in CPP could eliminate or modify the unpleasant seaweed smell of algal samples, which could broaden its application in the food and cosmetic industries. As reported by other investigators, the fucoidan content in *Sargassum* sp. (e.g., *S. pallidum*) ranged 7%–9% (dry basis) [21]. We obtained fucoidan extracts from the compressional-puffed algal samples by warm water extraction and ethanol precipitation. The extraction yields of fucoidan for SC1, SC2, and SC3 are presented in Table 1. The extraction yields for SC1, SC2, and SC3 were significantly increased (0.68 ± 0.07, 0.90 ± 0.04 to 1.08 ± 0.04 g/100 g, dry basis) (*p* < 0.05) by raising the puffing pressures from 0, 1.7 to 6.3 kg/cm^2^, indicating that CPP could effectively augment the extraction yield of fucoidan by damaging the algal plant matrices, thus allowing the solvent molecules to easily penetrate to the cytoplasm layer, resulting in a higher yield of fucoidan. The fucoidan extraction yields reported in the literature showed a wide range of values. Sinurat et al. extracted fucoidan from *S. binderi* using 0.01 M HCl at room temperature and found the extraction yield was 4.02% [22]. In addition, Wang et al. extracted fucoidan from *S. cristaefolium* using 40 °C double-distilled water and found the extraction yield was 0.73% (dry basis) [23]. It should be clearly noted that using chemicals to extract fucoidan resulted in a higher extraction yield than that achieved using water. However, excess usage of chemicals is problematic owing to their potential effects on the environment. Therefore, a combination of CPP and water extraction is recommended for the extraction of fucoidan. Taken together, our data showed that the CPP method could decrease the bulk density of algal samples, expand the algal cellular structures, eliminate the unpleasant algal odor, and augment the extraction yield of fucoidan in algal samples.

### 2.2. Physicochemical and Compositional Analyses of Fucoidans for SC1, SC2, and SC3

**Figure 2:**
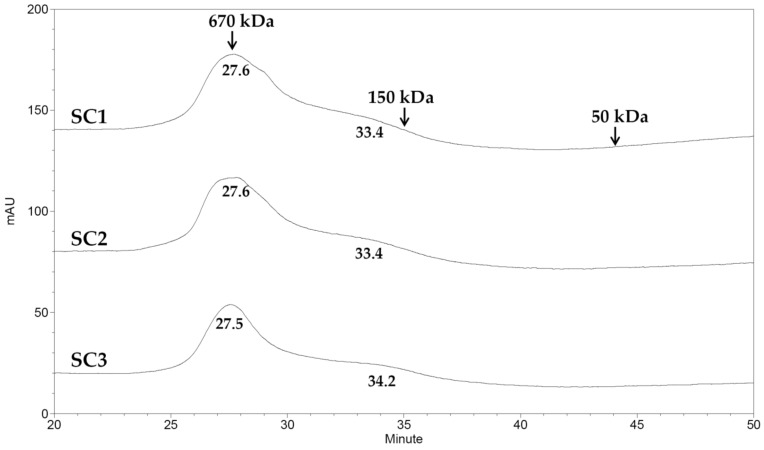
Size exclusion chromatographic profiles for SC1, SC2, and SC3. Dextrans with molecular weights 50, 150, and 670 kDa were utilized as the standards.

**Figure 3:**
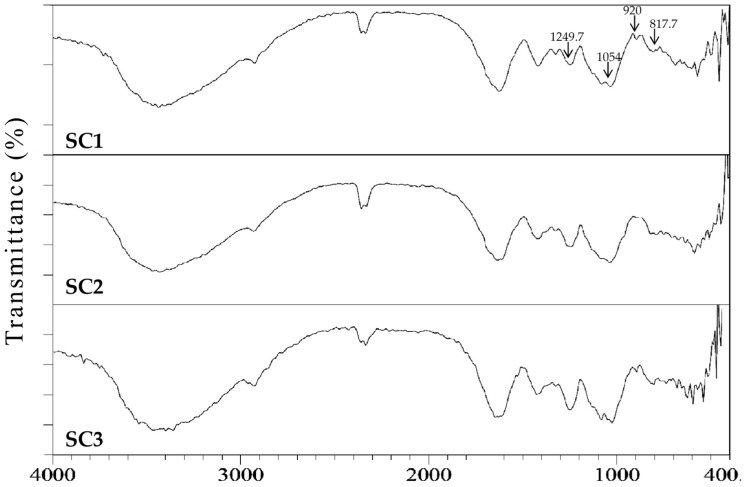
FTIR spectra for SC1, SC2, and SC3. Absorption bands at 817.7, 920, 1054, 1249.7 cm^−^^1^ are indicated.

The extracts obtained for SC1–SC3 were characterized with respect to the molecular weight, Fourier transform infrared (FTIR) spectroscopy, and compositional analyses. The high-performance liquid chromatography (HPLC) gel filtration analysis revealed that the averaged molecular weights of extracts for SC1 were 627.18 and 240.02 kDa; for SC2 were 628.97 and 237.26 kDa; and for SC3 were 641.20 and 209.35 kDa, respectively (Figure 2). The molecular weight pattern of these three extracts is similar, indicating that CPP did not obviously alter the molecular weight distribution in SC1–SC3. The FTIR results of SC1–SC3 showed typical signals for sulfated polysaccharides as well as the presence of sulfate groups (Figure 3). The peak at 817.7 cm^−1^ corresponds to the bending vibrations of C-O-S of sulfate [2], the peak at 920 cm^−1^ indicates β-pyranose ring vibration [24], the peak at 1054 cm^−1^ shows C-O-H vibration [24], and the peak at 1249.7 cm^−1^ indicates the asymmetric stretching vibration of sulfate group (S=O) [25]. Due to the similarity of the FTIR spectrums in SC1–SC3, it was deduced that the position of sulfate groups and structural aspects of sulfated polysaccharide were not significantly altered by CPP. The compositional analyses for SC1–SC3 were conducted and the results are shown in Table 2. The total sugar contents for SC1–SC3 ranged from 41.74% ± 1.26% to 56.41% ± 0.35% *(w/w*, dry basis), which were lower than the total sugar content (61%, dry basis) of polysaccharides generated from brown alga *S. tenerrimum* in a previous study [26]. A plausible explanation for this difference is that certain impurities such as protein, polyphenols, and alginate were co-extracted by our extraction procedure, which thus lowered the total sugar contents of SC1–SC3. A previous investigation suggested that polysaccharide with a higher uronic acid content may have higher antioxidant activity [27]. Here, we found that the contents of uronic acid for SC1, SC2, and SC3 were significantly increased from 12.68% ± 0.25%, 15.83% ± 0.90%, to 23.55% ± 1.99% (*p* < 0.05) as the puffing pressures elevated from 0, 1.7 to 6.3 kg/cm^2^, suggesting that CPP may facilitate the increase of uronic acid content in these fucoidans (Table 2). Algal fucoidan was reported to be an α- l-fucose-based polysaccharide [28]. The fucose contents for SC1, SC2, and SC3 were thus analyzed and the results were 28.77% ± 2.02%, 25.06% ± 1.75%, and 31.09% ± 1.00%, respectively, suggesting that CPP did not affect fucose content (Table 2). Previous studies suggested that the sulfate content of fucoidan has a crucial role in its biological functions [29,30]. Therefore, we measured the sulfate contents for SC1, SC2, and SC3 and the data were 23.84% ± 0.08%, 23.59% ± 0.41%, and 22.08% ± 0.55%, respectively (Table 2). It is notably apparent that there was no difference in sulfate content among SC1–SC3. However, a previous investigation has also suggested that the molar ratio of sulfate/fucose in fucoidan might play an important role in bioactivities, including antioxidant and anticoagulant activities [31]. We thus conducted a further analysis and found that the molar ratios of sulfate/fucose in SC1, SC2, and SC3 were 1.42 ± 0.09, 1.62 ± 0.10, and 1.22 ± 0.04, respectively (Table 2), and SC2 had the highest value of molar ratio of sulfate/fucose among SC1–SC3. Thus, it was expected that SC2 may exhibit high biological activity, and therefore further investigation is warranted. In addition, previous investigations indicated that crude extracts of fucoidan may contain a lot of impurities such as proteins, phenolic compounds, and alginic acids, which are hard to get rid of [32,33]. We found that a higher impurity of polysaccharides caused by the presence of protein, polyphenols, and alginate was detected in SC1 (5.08 + 3.52 + 9.54 = 18.14, g/100 g, dry basis), followed by SC2 (3.05 + 2.63 + 9.60 = 15.28, g/100 g, dry basis), and the least impurity of polysaccharides was detected in SC3 (2.79 + 2.77 + 8.84 = 14.40, g/100 g, dry basis) (Table 2), indicating that although CPP cannot completely remove impurities such as protein, polyphenols, and alginate, it may potentially decrease the amount of impurities in extracts of fucoidan, which would offer an advantage for commercial production of fucoidan with a high level of purity. These results are also consistent with our previous analyses of *S. glaucescen* [13]. Additionally, the monosaccharide compositions of SC1–SC3 were measured and the results are shown in Table 2. It was found that the dominant monosaccharides in SC1–SC3 were fucose and galactose, as well as smaller amounts of glucuronic acid, mannose, rhamnose, and xylose. In general, the monosaccharide composition of fucoidan is reported to vary considerably due to species differences, anatomical region, growing conditions, extraction procedures, and even the analytical methods used [34]. Although different puffing pressures were introduced in SC1–SC3, we found that the monosaccharide compositions of SC1–SC3 were not apparently altered. Overall, the SC1–SC3 showed characteristics of fucoidan as illustrated by the molecular weight, FTIR spectroscopy, and compositional analyses. CPP had beneficial effects in terms of the increased uronic acid content and decreased impurities of polysaccharides. Nevertheless, the molecular weight, FTIR signals, total sugar content, fucose content, sulfate content, and monosaccharide composition of fucoidan were not notably influenced by CPP. In addition, SC2 contained the highest molar ratio of sulfate/fucose as compared to SC1 and SC3, and thus the biological function of SC2 warrants further examination.

### 2.3. Antioxidant Activities of SC1, SC2, and SC3

**Figure 4:**
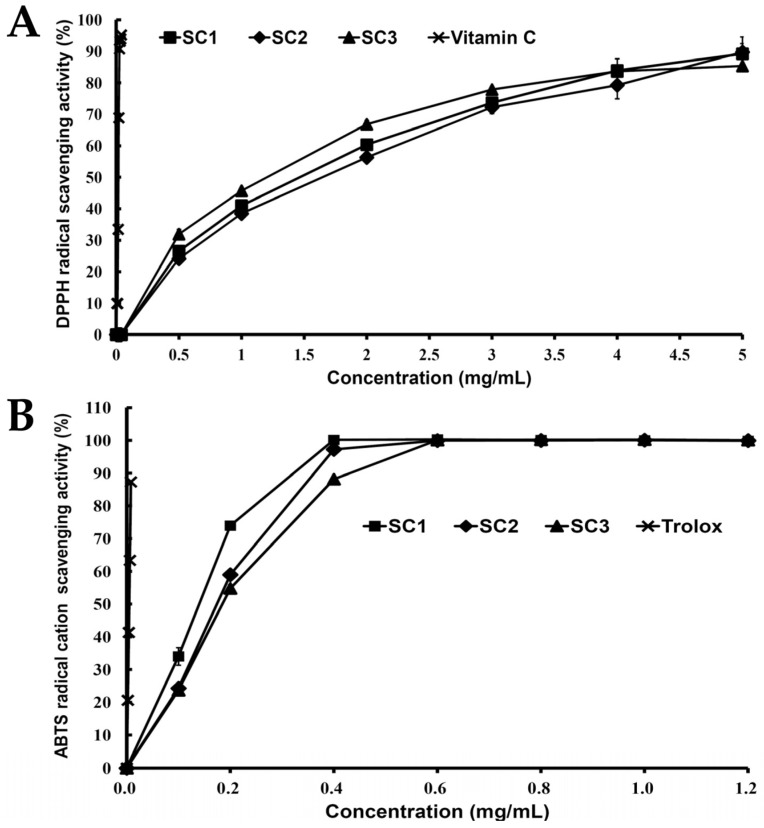
Antioxidant activities of crude extracts of fucoidan (SC1, SC2, and SC3): (**A**) DPPH radical scavenging activity for SC1, SC2, SC3, and vitamin C. Values are expressed as the mean ± SD (n = 3); and (**B**) ABTS radical cation scavenging activity for SC1, SC2, SC3, and Trolox. Values are expressed as the mean ± SD (n = 3).

The antioxidant activities of SC1–SC3 were examined by 2,2-diphenyl-1-picrylhydrazyl (DPPH) and 2,2’-azino-bis(3-ethylbenzothiazoline-6-sulphonic acid) diammonium salt (ABTS) methods. DPPH is known to be a stable free radical and is widely used to evaluate the antioxidant activity in a relatively short time compared to other methods [31]. Figure 4A shows the DPPH radical scavenging properties of SC1, SC2, SC3, and vitamin C (as a reference). It can be seen that SC1–SC3 displayed DPPH radical scavenging activity in a dose-dependent pattern. ABTS^•^^+^ is a long-lived cation free radical, which is decolorized during the reaction with hydrogen-donating antioxidant [35]. The ABTS^•^^+^ scavenging properties of SC1, SC2, SC3, and 6-hydroxy-2,5,7,8-tetramethylchroman-2-carboxylic acid (Trolox) (as a reference) are presented in Figure 4B. All samples (SC1–SC3) showed ABTS^•^^+^ scavenging activity in a dose-dependent manner. In addition, previous investigations have demonstrated that oxidative stress-induced cell damage was implicated both in the physiological process of aging and in neurodegenerative diseases [3,4]. Thus, the antioxidant activities of SC1–SC3 warrant further study to fully elucidate their neuroprotective functions.

### 2.4. Neuroprotective Activities of SC1, SC2, and SC3

**Figure 5:**
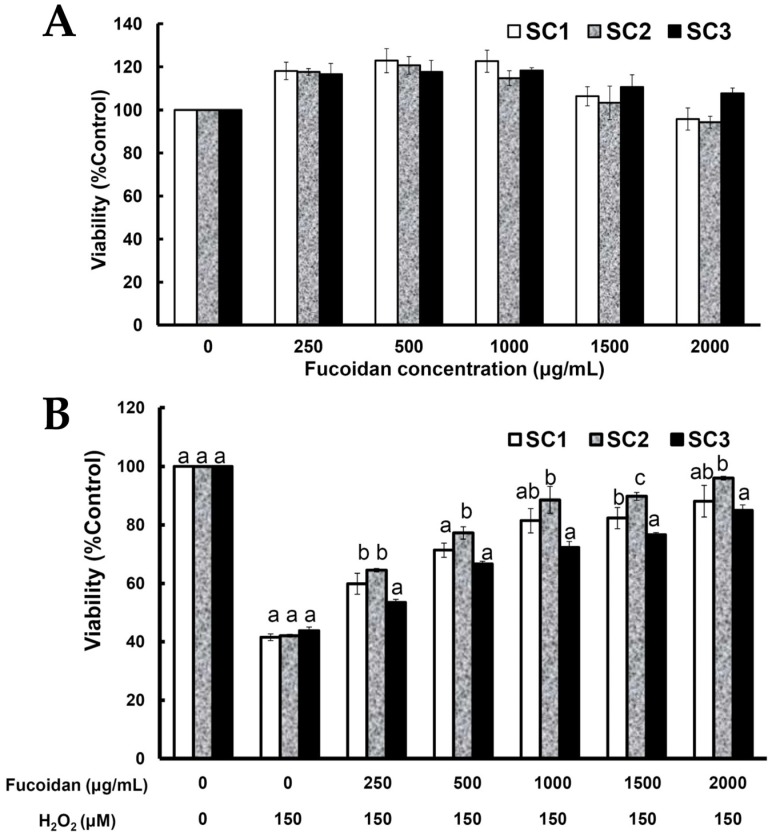
Effects of crude extracts of fucoidan (SC1, SC2, and SC3) and H_2_O_2_ treatment with or without SC1–SC3 pretreatment on cell viability of PC-12 cells: (**A**) PC-12 cells were treated with SC1, SC2, or SC3 (0–2000 μg/mL) for 24 h, and cell viability was assessed. Values are expressed as the mean ± SD (n = 3); (**B**) PC-12 cells were pretreated with SC1, SC2, or SC3 (0–2000 μg/mL) for 24 h, followed by treatment with 150 μM H_2_O_2_ for 30 min, and cell viability was assessed. Values are expressed as the mean ± SD (n = 3). In each group of columns related to each concentration of fucoidan, the means that have at least one common letter do not differ significantly (*p* < 0.05).

**Figure 6:**
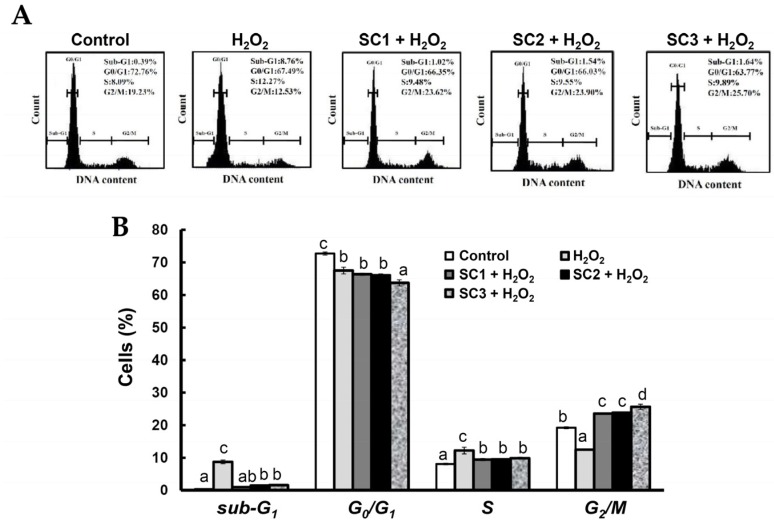
Effects of H_2_O_2_ treatment with or without SC1–SC3 pretreatment on cell cycle profiles of PC-12 cells: (**A**) PC-12 cells were pretreated with SC1, SC2, or SC3 at a concentration of 2000 μg/mL for 24 h, followed by treatment with 150 μM H_2_O_2_ for 4.5 h, and cell cycle profiles were assessed; (**B**) the bar graph summarizes the three cell cytometry experiments and shows the percentages of cells in the *sub-G_1_*, *G_0_/G_1_*, *S*, and *G_2_/M* phase of the cell cycle according to treatments after analysis using BD Accuri C6 software. Values are expressed as the mean ± SD (n = 3). In each group of columns related to each cell cycle phase, the means that have at least one common letter do not differ significantly (*p* < 0.05).

**Figure 7:**
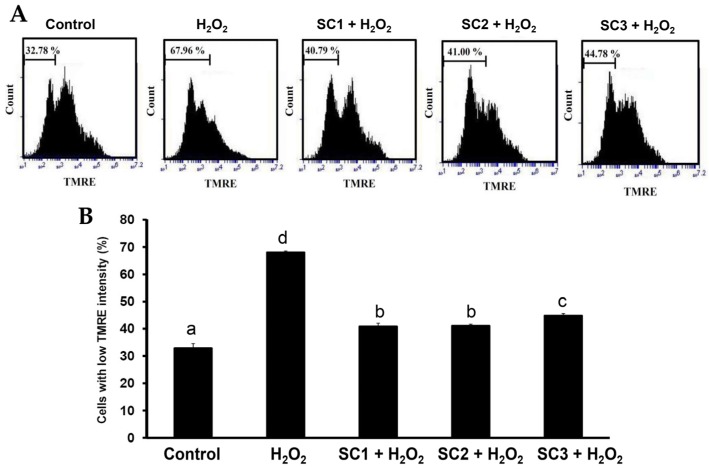
Effects of H_2_O_2_ treatment with or without SC1–SC3 pretreatment on MMP analysis of PC-12 cells: (**A**) PC-12 cells were pretreated with SC1, SC2, or SC3 at a concentration of 2000 μg/mL for 24 h, followed by treatment with 150 μM H_2_O_2_ for 4.5 h, and MMP was determined by TMRE staining and flow cytometry; (**B**) the bar graph summarizes the three cell cytometry experiments and shows the percentage of cells with low TMRE intensity according to treatments after analysis using BD Accuri C6 software. Values are expressed as the mean ± SD (n = 3). The means that have at least one common letter do not differ significantly (*p* < 0.05).

**Figure 8:**
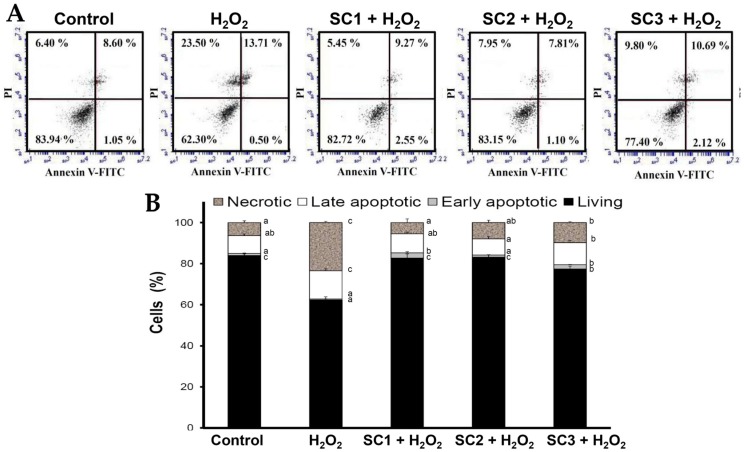
Effects of H_2_O_2_ treatment with or without SC1–SC3 pretreatment on annexin V-FITC/PI double staining analysis of PC-12 cells: (**A**) PC-12 cells were pretreated with SC1, SC2, or SC3 at a concentration of 2000 μg/mL for 24 h, followed by treatment with 150 μM H_2_O_2_ for 4.5 h, and annexin V-FITC/PI double staining analysis was performed by flow cytometry; and (**B**) the bar graph summarizes the three cell cytometry experiments and shows the percentages of living, early apoptotic, late apoptotic, and necrotic cells according to treatments after analysis using BD Accuri C6 software. Values are expressed as the mean ± SD (n = 3). In each group of columns related to each cell population, the means that have at least one common letter do not differ significantly (*p* < 0.05).

Since rat pheochromocytoma PC-12 cells can act as a neuron-like system and can also be used to monitor cell death due to oxidative stress [36], we used PC-12 cells to study the rescue effects of SC1–SC3 on H_2_O_2_-induced cellular impairment. To evaluate the cytotoxic effect of SC1–SC3 on PC-12 cells, the cells were treated with different concentrations of SC1–SC3 for 24 h, and then the cell viability of PC-12 cells was evaluated by Alamar Blue assay. As shown in Figure 5A, at concentrations from 0 to 2000 μg/mL, none of the studied extracts SC1–SC3 exhibited cytotoxicity to PC-12 cells. The treatment of PC-12 cells with 150 μM H_2_O_2_ for 30 min decreased the cell viability and the value was 41.53%–43.75% of the control group (Figure 5B). Moreover, pretreatment of PC-12 cells with SC1–SC3 at concentrations of 250–2000 μg/mL for 24 h dose-dependently attenuated H_2_O_2_-induced cellular cytotoxicity (Figure 5B). A further calculation revealed that the maximum recovery ratios (the highest cell viability/the lowest cell viability) for SC1, SC2, and SC3 were 2.12 (88.10/41.53 = 2.12), 2.28 (95.99/42.07 = 2.28), and 1.94 (84.98/43.75 = 1.94), respectively. Thus, SC2 had the greatest effect on reversion of H_2_O_2_-induced cytotoxicity, followed by SC1, and then SC3. Interestingly, this trend was similar to the finding indicated in Table 2 that shows SC2 had the highest molar ratio of sulfate/fucose, followed by SC1, and then SC3. Thus, it appears that molar ratio of sulfate/fucose in fucoidan may play an important role in its biological functions such as attenuation of oxidative stress-induced cellular cytotoxicity. However, further in vitro experiments such as oversulfation of fucoidan and in vivo studies are necessary to explore the mechanisms involved. H_2_O_2_ is known to be a major source of ROS which may destroy neurons by inducing apoptosis [37]. In order to further examine the protective effects of SC1–SC3 on H_2_O_2_-induced neuronal cellular apoptosis, the cell cycle distribution, mitochondrial membrane potential (MMP), and annexin V-fluorescein isothiocyanate (FITC) assays were performed by flow cytometry. When cells undergo propidium iodide (PI) staining, the flow cytometry can detect apoptotic dead cells as well as those with fragmented nuclei, which are also called *sub-G_1_* cells [38]. As shown in Figure 6A,B, analysis of DNA contents following 150 μM H_2_O_2_ treatment of PC-12 cells revealed a significant increase in the proportion of cells with *sub-G_1_* DNA content to 8.76% ± 0.57% as compared to untreated cells (0.39% ± 0.12%) (*p* < 0.05). Treatment of cells with 150 μM H_2_O_2_ in the presence of 2000 μg/mL SC1, SC2, or SC3 significantly reduced the apoptotic *sub-G_1_* populations to 1.02% ± 0.17%, 1.54% ± 0.26%, and 1.64% ± 0.04%, respectively (*p* < 0.05). These findings suggest that the PC-12 cells exposed to H_2_O_2_ exhibited an obvious increase in the percentage of cells with *sub-G_1_* DNA content, which indicates enriched DNA fragmentation and apoptosis in the cells. Moreover, treatment of cells with SC1, SC2, or SC3 significantly reduced the *sub-G_1_* populations of H_2_O_2_-induced apoptotic cells, suggesting that SC1, SC2, and SC3 had a protective effect on PC-12 against oxidative damage. When PC-12 cells were treated with 150 μM H_2_O_2_, entry into the *S* phase of the cell cycle was arrested or delayed (12.27% ± 1.03%) as compared to untreated cells (8.09% ± 0.23%) (Figure 6B). Furthermore, treating cells with 150 μM H_2_O_2_ in the presence of 2000 μg/mL SC1, SC2, or SC3 attenuated the *S* phase populations to 9.48% ± 0.34%, 9.55% ± 0.07%, and 9.89% ± 0.29%, respectively, suggesting that SC1, SC2, and SC3 may diminish H_2_O_2_-induced growth inhibition of PC-12 cells. A previous investigation demonstrated that ROS can affect mitochondrial function through the mitochondrial adenosine 5′-triphosphate (ATP)-sensitive potassium (mito KATP) channels and the mitochondrial permeability transition pore (mPTP). The irreversible opening of mPTP is indicative of early apoptosis and is lethal to cells [39]. Maintenance of MMP is necessary for production of energy (ATP) and preservation of cellular homeostasis [40]. Thus, the loss of MMP is a hallmark of apoptosis and is directly linked to the induction of apoptosis [41]. To quantify loss of MMP, a potentiometric fluorescent tetramethylrhodamine ethyl ester (TMRE) dye can be used. As TMRE is a cell-permeable, positively-charged, red-orange dye that readily accumulates in active mitochondria due to their relative negative charge, the mitochondrial function can be assessed by examining the TMRE accumulation in mitochondria. As shown in Figure 7A,B, the percentage of TMRE negative cell number in the untreated sample was 32.78% ± 1.80%. When PC-12 cells were treated with 150 μM H_2_O_2_, the percentage of TMRE negative cell number was significantly increased to 67.96% ± 0.59% (*p* < 0.05), which suggests that H_2_O_2_ could cause membrane depolarization and thus decrease the accumulation of TMRE. In contrast, when cells were treated with 150 μM H_2_O_2_ in the presence of 2000 μg/mL SC1, SC2, or SC3, the percentages of TMRE negative cell numbers were decreased to 40.79% ± 1.27%, 41.00% ± 0.68%, and 44.78% ± 0.81%, respectively. These results clearly indicate that SC1–SC3 may protect PC-12 cells from H_2_O_2_-induced mitochondrial dysfunction. It has been shown that the loss of plasma membrane asymmetry is an early event in apoptosis, resulting in the exposure of phosphatidylserine (PS) residues at the outer plasma membrane [42]. Annexin V was shown to interact strongly and specifically with PS and can be used to detect apoptosis by targeting loss of plasma membrane integrity [42]. Thus, annexin V staining is a feasible tool to detect apoptosis in the early stage. In the present study, we used an annexin V-FITC and PI double-staining method to analyze early or late stage apoptosis as well as necrotic cells. Viable cells with intact membranes exclude PI, whereas the membranes of dead and damaged cells are permeable to PI. For instance, viable cells are annexin V-FITC and PI negative, and cells that are in early apoptosis are annexin V-FITC positive and PI negative. Cells in late apoptosis are both annexin V-FITC and PI positive, and cells that are in necrosis are annexin V-FITC negative and PI positive. As shown in Figure 8A,B, exposure of PC-12 cells to 150 μM H_2_O_2_ generally resulted in an increase in the percentages of late apoptotic and necrotic cells, and a reduction in the percentage of live cells as compared to untreated cells. When cells were treated with 150 μM H_2_O_2_ in the presence of 2000 μg/mL SC1, SC2, or SC3, the percentages of late apoptotic and necrotic cells were obviously decreased, and the percentage of live cells was increased as compared to H_2_O_2_-treated cells. These findings clearly indicate that SC1–SC3 had a protective effect on PC-12 cells against H_2_O_2_-induced cell death (consisting of apoptosis and necrosis). In summary, all of the treated extracts SC1–SC3 may protect PC-12 cells from H_2_O_2_-induced cell death (i.e., apoptosis and necrosis) as illustrated by cytotoxicity, cell cycle distribution, MMP, and annexin V-FITC analyses. The molar ratio of sulfate/fucose in fucoidan seemed to be associated with its biological functions such as attenuation of oxidative stress-induced cellular cytotoxicity. In addition, although both SC1 and SC2 possess high and similar neuronal protective properties, SC2 showed a higher extraction yield, higher efficacy in the reversion of H_2_O_2_-induced cytotoxicity in PC-12 cells, and a lower level of impurities compared with SC1, and thus SC2 is suggested as a good candidate for future development as a therapeutic agent for preventive treatment of neurodegenerative diseases.

## 3. Materials and Methods

### 3.1. Materials and Chemicals

A sample of *S. crassifolium* (SC), collected from Kenting (Pingtung, Taiwan), was washed with fresh water soon after collection in order to remove salt and sand, oven-dried at 50 °C, and then kept in plastic bags at 4 °C until use. l-fucose, D-glucuronic acid, D-galacturonic acid, gallic acid, sodium carbonate, potassium sulfate, DPPH, ABTS, Trolox, and dimethyl sulfoxide (DMSO) were purchased from Sigma-Aldrich (St. Louis, MO, USA). Potassium bromide (KBr), potassium persulfate, and sodium sulfite were purchased from Merck (Darmstadt, Germany). Alamar blue was purchased from Invitrogen (Carlsbad, CA, USA). 2,2,2-Trifluoroacetic acid (TFA) was obtained from Panreac (Barcelona, Spain). RPMI-1640 medium, trypsin/EDTA, fetal bovine serum (FBS), penicillin, and streptomycin were purchased from Gibco Laboratories (Grand Island, NY, USA). All other chemicals used were obtained from Sigma-Aldrich (St. Louis, MO, USA) and were all of analytical grade.

### 3.2. Compressional-Puffing Procedure

The dried algal sample was crumbled and sieved using a 20-mesh screen. The portion retained by the screen was collected and then puffed according to a previously described procedure [13] with slight modification. In brief, the algal sample (weight = 2.5 g, H_2_O = 8.1%) was automatically put into the chamber and the puffing conditions were set with temperatures 140 and 180 °C, respectively (Table 1). After CPP, the algal sample was ground into fine particles and stored at 4 °C for further extraction experiments.

### 3.3. Warm Water Extraction Procedure

The compressional-puffed algal sample was mixed with 95% ethanol (*w/v* = 1:10), shaken for 4 h at room temperature to remove pigments, proteins and lipid, and then centrifuged at 970× *g* for 10 min. The residue was collected, mixed with double-distilled water (*w/v* = 1:10) and placed in a water-bath kept at 40 °C for 15 min with shaking (120 rpm) to extract the polysaccharides. The mixture was centrifuged at 3870× *g* for 10 min and the supernatant was collected. Ethanol (95%) was added into the supernatant to give a final ethanol concentration of 20% in order to precipitate alginic acid. The mixture was centrifugated at 9170× *g* for 30 min, the supernatant was collected, and 95% ethanol was added until a final ethanol concentration of 50% was reached in order to obtain fucoidan precipitate. The ethanol-precipitated fucoidan was then recovered by centrifugation at 9170× *g* for 30 min, dried at 40 °C, milled, and stored for further analyses. Extraction yield was calculated using the following equation:

Extraction yield (%) = (*g*_A_/*g*_B_) × 100 (1)

where *g*_A_ represents the weight of the extracted solid on a dry basis, and *g*_B_ is the weight of the sample on a dry basis.

### 3.4. Analytical Methods

The determinations of crude protein, fat, moisture, and ash were carried out using the following AOAC procedures. Protein, fat, and ash were calculated on a dry basis. Carbohydrate was calculated as weight differences between the total weight and the sum of the amounts of moisture, protein, lipid, and ash. The phenol-sulfuric acid colorimetric method was utilized to determine the total sugar content, and l-fucose was used as the standard. The fucose content was determined according to the method of Gibbons [43] using l-fucose as the standard. Uronic acids were estimated by the colorimetric method using d-galacturonic acid as the standard [44]. Protein in the extract was quantified by the Bradford method using BSA as the standard. Polyphenols were analyzed by the Folin-Ciocalteu method and gallic acid was utilized as a standard agent. Alginate content was measured according to the previously described method [45]. For the determination of sulfate content, the sample was hydrolyzed with 1 N HCl solution for 5 h at 105 °C. The hydrolysate was then quantified based on the percentage of sulfate composition using Dionex ICS-1500 Ion Chromatography (Sunnyvale, CA, USA) with an IonPac AS9-HC column (4 × 250 mm) at a flow rate of 1 mL/min at 30 °C with conductometric detection. A solution of 9 mM Na_2_CO_3_ was used as the eluent, and K_2_SO_4_ was used as the standard.

### 3.5. Color Analysis

The algal samples (about 15 g) with different puffing conditions were used for the determination of color. Tristimulus color values, namely *L* (lightness), *a* (redness-greenness), and *b* (yellowness-blueness) values, were measured using a spectrophotometer (SA-2000, Nippon Denshoku Industries Co., Ltd., Tokyo, Japan). The data were recorded using at least three separate algal samples for each test point. The blackness was obtained using the following equation:

(2) $$\text{Blackness }=\sqrt{{\left(100-L\right)}^{2}\;+\;a^{2}\;+\;b^{2}}$$

### 3.6. Sensory Evaluation

Sensory evaluation was conducted using a 30-member panel. Algal samples (about 30 g) from different puffing conditions were placed in test tubes with screw caps. Panelists were instructed to remove the screw caps, smell the contents, and identify the odor that they perceived, as well as indicate their degree of liking of the odor. The degree of liking was based on a seven-point hedonic scale (1 = dislike extremely; 4 = neither like nor dislike; 7 = like extremely).

### 3.7. Bulk Density

The volume of the algal samples with different puffing conditions was determined by filling a container of known volume, and noting the sample weight. The bulk density was calculated as the ratio of the mass of the sample to that of its volume. Bulk density results were based on an average of five measurements.

### 3.8. Analysis of Monosaccharide Composition

The polysaccharide samples were hydrolyzed with 4 N TFA at 110 °C for 4 h. After removing the residual acid, the hydrolysates were then utilized to determine the monosaccharide composition using Dionex ICS-1500 Ion Chromatography with Dionex CarboPac SA10 column (4 × 250 mm) at a flow rate of 1.5 mL/min at 30 °C with conductometric detection. A solution of 10 mM NaOH was used as the eluent, and l-fucose, d-xylose, d-galactose, d-glucose, d-glucuronic acid, l-rhamnose, and d-mannose were used as the standards.

### 3.9. Molecular Weight Analysis

The molecular weight analysis of the polysaccharides was conducted with a size exclusion HPLC column Superdex 200 (300 mm × 10 mm ID, GE Healthcare, Piscataway, NJ, USA) using a SHIMADZU HPLC system (Shimadzu, Kyoto, Japan) equipped with a refractive index detector. The chromatography conditions were: eluent 0.2 M NaCl; flow rate 0.3 mL/min, sample concentration 10 mg/mL; injection volume 0.1 mL; and temperature 25 °C. The standards used to calibrate the column were various dextrans with different molecular weights (50, 150, and 670 kDa) (Sigma-Aldrich, St. Louis, MO, USA).

### 3.10. Scanning Electron Microscopy (SEM) Examination

The morphological characteristics of algal samples were observed by SEM. Dehydrated algal tissues from blade and stem were first coated with gold via a sputter-coater at ambient temperature. After coating with gold, the algal surfaces were examined with a JEOL JSM-5300 (Peabody, MA, USA) scanning electron microscope at 5 kV.

### 3.11. Fourier Transform Infrared (FTIR) Spectroscopy

Two mg of polysaccharides were ground evenly with approximately 100 mg KBr until particles measured < 2.5 μm in size. The transparent KBr pieces were made at 500 kg/cm^2^ under vacuum. The FTIR spectra were obtained using a FT-730 spectrometer (Horiba, Kyoto, Japan). The signals were automatically collected using 60 scans over the range of 4000–400 cm^−^^1^ at a resolution of 16 cm^−^^1^ and were compared to a background spectrum collected from the KBr alone at room temperature [46].

### 3.12. DPPH Radical Scavenging Activity

The scavenging activity of the DPPH radical in the samples was determined using a previously method described [23]. In brief, 50 µL of sample was added to 150 µL 0.1mM DPPH solution (in methanol). The mixture was shaken vigorously for 1 min and left to stand for 30 min in the dark at room temperature. After the reaction, the absorbance of all sample solutions was then measured at 517 nm using an ELISA reader (PowerWave 340, Bio-Tek Instruments, Winooski, VT, USA). The radical-scavenging activity was calculated as the percentage inhibition using the following equation:

(3) $${\mathrm{DPPH}}_{\mathrm{radical-scavenging}}(\%)=[1-\frac{A_{\mathrm{sample}}}{A_{\mathrm{control}}}\;]\times 100$$

where A_sample_ is the absorbance of the methanol solution of DPPH with tested samples, and A_control_ represents the absorbance of the methanol solution of DPPH without the sample.

### 3.13. ABTS Radical Cation Scavenging Activity

The ABTS radical cation scavenging activity was performed according to the method [23]. The ABTS^•^^+^ solution was produced by mixing 5 mL of 7 mM ABTS solution with 88 µL of 140 mM potassium persulfate and allowing the mixture to stand in the dark for 16 h at room temperature before use. The ABTS^•^^+^ solution was diluted with 95% ethanol so that its absorbance at 734 nm was adjusted to 0.70 ± 0.05. To determine the scavenging activity, 100 µL diluted ABTS^•^^+^ solution was mixed with 100 µL of various sample solutions and the mixture was allowed to react at room temperature for 6 min. After the reaction, the absorbance of all sample solutions was then measured at 734 nm using an ELISA reader (PowerWave 340, Bio-Tek Instruments, Winooski, VT, USA). The blank was prepared in the same manner, except that distilled water was used instead of the sample. The scavenging activity of ABTS^•^^+^ was calculated using the following equation:

(4) $${\mathrm{ABTS}}_{\mathrm{cation radical-scavenging}}(\%)=\left[1-\frac{A_{\mathrm{sample}}}{A_{\mathrm{control}}}\;\right]\times 100$$

where A_sample_ is the absorbance of ABTS with tested samples, and A_control_ represents the absorbance of ABTS without the sample.

### 3.14. Cell Culture

Rat pheochromocytoma PC-12 (ATCC^®^ CRL-1721™) cells were purchased from Food Industry Research and Development Institute, Hsinchu, Taiwan which had originally obtained them from American Type Culture Collection (ATCC). PC-12 cells were cultured according to ATCC instructions. These include PC-12 cells grown in RPMI 1640 medium supplemented with heat-inactivated at 56 °C, 10% horse serum, and 5% FBS, plus 1% penicillin/streptomycin antibiotics at 37 °C in the presence of 95% air, and 5% CO_2_ in T-75 cell culture flasks (Corning, Corning, NY, USA). The culture medium was changed every 48 h.

### 3.15. Cell Viability Test

Alamar Blue assay was applied to test the cell viability. In brief, cells were plated at 3.3 × 10^5^ cells/mL in 96-well plates with 100 µL RPMI-1640 growth medium and incubated for 24 h at 37 °C, with 5% CO_2_ in a humidified atmosphere. The cells were then exposed to test compounds at various concentrations for various times. Thereafter, the reaction of tested compounds was stopped by removing the treatment media from each well. Then growth media containing 10% Alamar Blue reagent was added to each well, and the plates were allowed to incubate at 37 °C in 5% CO_2_ for 4 h. After the incubation, the absorbance at 570 and 600 nm was measured with an ELISA reader (PowerWave 340, Bio-Tek Instruments, Winooski, VT, USA). The cell viability was determined according to manufacturer’s instructions (Invitrogen, Carlsbad, CA, USA).

### 3.16. Cell Cycle Analysis

Cells were plated at 1 × 10^6^ cells/mL in 24-well plates with RPMI-1640 growth medium and incubated for 24 h at 37 °C, with 5% CO_2_ in a humidified atmosphere. Thereafter, cells were pretreated with different fucoidans at a final concentration of 2000 μg/mL for 24 h, and then exposed to H_2_O_2_ at a final concentration of 150 μM for 4.5 h. Floating and adherent cells were then collected and washed with 1× ice-cold phosphate-buffered saline (PBS) twice. The cell pellets were collected and fixed with 70% ice-cold ethanol and then stored in the freezer for at least 2 h. After washing with staining buffer twice, cells were then stained with 50 μg/mL PI in the presence of 25 μg/mL RNase A at 37 °C for 15 min. Flow cytometric analysis was completed using a BD Accuri C6 flow cytometer (BD Biosciences, San Jose, CA, USA) and a minimum of 10,000 cells per sample was collected. To estimate the percentage of each phase in the cell cycle, the data were analyzed by BD Accuri C6 software.

### 3.17. Mitochondrial Membrane Potential (MMP) Analysis

Cells were plated at 1 × 10^6^ cells/mL in 24-well plates with RPMI-1640 growth medium and incubated for 24 h at 37 °C, with 5% CO_2_ in a humidified atmosphere. Thereafter, cells were pretreated with different fucoidans at a final concentration of 2000 μg/mL for 24 h, and then exposed to H_2_O_2_ at a final concentration of 150 μM for 4.5 h. Floating and adherent cells were then collected, and washed with 1× ice-cold PBS twice. The cell pellets were collected and the cell density was adjusted to 1 × 10^6^ cells/mL with staining buffer and then incubated with 100 nM TMRE (Molecular Probes, Invitrogen Corp., Carlsbad, CA, USA) in the dark for 15–30 min at 37 °C. After the reaction, TMRE was removed by centrifugation. The cells were then washed with staining buffer twice, resuspended in staining buffer, and analyzed by BD Accuri C6 flow cytometer (BD Biosciences, San Jose, CA, USA) and a minimum of 10000 cells per sample were collected. The data were analyzed by BD Accuri C6 software.

### 3.18. Annexin V-Fluorescein Isothiocyanate (FITC) Staining Analysis

Cells were plated at 1 × 10^6^ cells/mL in 24-well plates with RPMI-1640 growth medium and incubated for 24 h at 37 °C, with 5% CO_2_ in a humidified atmosphere. Thereafter, cells were pretreated with different fucoidans at a final concentration of 2000 μg/mL for 24 h, and then exposed to H_2_O_2_ at a final concentration of 150 μM for 4.5 h. Floating and adherent cells were then collected, and washed with 1× ice-cold PBS twice. The cell pellets were collected and the cell density was adjusted to 1 × 10^5^ cells/100 µL with binding buffer (10 mM HEPES/NaOH (pH 7.4), 140 mM NaOH, and 2.5 mM CaCl_2_), and supplemented with 5 µL of annexin V-FITC (BD PharMingen, San Diego, CA, USA) and 5 µL of PI, then incubated in the dark for 15 min at 25 °C. The samples were analyzed using a BD Accuri C6 flow cytometer (BD Biosciences, San Jose, CA, USA) within 1 h and a minimum of 10000 cells per sample were collected. The data were analyzed by BD Accuri C6 software.

### 3.19. Statistical Analysis

Experiments were performed at least three times. Values represent the means ± standard deviation (SD). Statistical analyses were done using the Statistical Package for the Social Sciences (SPSS). The results obtained were analyzed using one-way analysis of variance (ANOVA), followed by Duncan’s Multiple Range tests. A probability value of *p* < 0.05 was considered statistically significant.

## 4. Conclusions

In this paper, we extracted fucoidan from *S. crassifolium* using a compressional puffing process (CPP) followed by warm water extraction. CPP decreased the bulk density of the algal sample, expanded the algal cellular structure, eliminated the unpleasant algal odor, increased the extraction yield, and decreased the impurities in fucoidan. All extracts (SC1–SC3) showed characteristics of fucoidan by compositional, FTIR spectroscopy, and molecular weight analyses. All extracts SC1–SC3 showed antioxidant activities dose-dependently. Although both SC1 and SC2 possessed high and similar neuronal protective properties, SC2 showed a higher extraction yield, higher efficacy on the reversion of H_2_O_2_-induced cytotoxicity in PC-12 cells, and a lower level of impurities compared with SC1, and thus SC2 may have potential for further development as a candidate neuroprotective agent. Future in vivo studies of SC2 as a possible therapeutic agent for preventive treatment of neurodegenerative diseases are warranted.

## Figures and Tables

**Table 1:** Compressional-puffing parameters, characteristics of puffed algal samples, and extraction yields of fucoidan extracts for SC1 (puffing at 0 kg/cm^2^), SC2 (puffing at 1.7 kg/cm^2^), and SC3 (puffing at 6.3 kg/cm^2^).

**Variables of Compressional-Puffing**		**SC1**	**SC2**	**SC3**
Mechanical compression	Pressure (kg/cm^2^)	0	5	5
	Number of compression times	0	3	3
Puffing	Temperature (°C)	0	140	180
	Pressure (kg/cm^2^)	0	1.7	6.3
Reaction time	Time (s)	0	10	10
**Variables of Water Extraction**		**SC1**	**SC2**	**SC3**
Extraction temperature	Temperature (°C)	40	40	40
Extraction time	Time (min)	15	15	15
**Characteristics of Puffed Algal Samples**		**SC1 ^1^**	**SC2 ^1^**	**SC3 ^1^**
Degree of moisture loss (%)		0.00 ± 0.00 ^a^	14.11 ± 2.36 ^b^	34.69 ± 6.02 ^c^
*L*		41.73 ± 0.00 ^c^	41.66 ± 0.00 ^b^	41.38 ± 0.00 ^a^
*a*		4.44 ± 0.00 ^c^	4.24 ± 0.01 ^b^	4.11 ± 0.00 ^a^
*b*		3.55 ± 0.04 ^b^	3.36 ± 0.02 ^a^	3.35 ± 0.02 ^a^
Blackness		58.54 ± 0.01 ^a^	58.59 ± 0.00 ^b^	58.86 ± 0.01 ^c^
Bulk density (g/mL)		0.54 ± 0.00 ^c^	0.52 ± 0.00 ^b^	0.50 ± 0.00 ^a^
Hedonic scores of algal odor		2.67 ± 0.79 ^a^	3.70 ± 0.97 ^b^	4.60 ± 0.84 ^c^
**Extraction Yields of Fucoidan**		**SC1 ^1^**	**SC2 ^1^**	**SC3 ^1^**
Extraction yield (%)		0.68 ± 0.07 ^a^	0.90 ± 0.04 ^b^	1.08 ± 0.04 ^c^

**^1^** Values are mean ± SD (*n* = 3); values in the same row with different letters (in ^a^, ^b^, and ^c^) are significantly different (*p* < 0.05).

**Table 2:** Compositional analyses for SC1, SC2, and SC3.

Chemical Composition	SC1 ^2^	SC2 ^2^	SC3 ^2^
Total sugar (%)	46.43 ± 0.80 ^b^	41.74 ± 1.26 ^a^	56.41 ± 0.35 ^c^
Uronic acid (%)	12.68 ± 0.25 ^a^	15.83 ± 0.90 ^b^	23.55 ± 1.99 ^c^
Fucose (%)	28.77 ± 2.02 ^a b^	25.06 ± 1.75 ^a^	31.09 ± 1.00 ^b^
Sulfate (%)	23.84 ± 0.08 ^a^	23.59 ± 0.41 ^a^	22.08 ± 0.55 ^a^
Protein (%)	5.08 ± 0.32 ^b^	3.05 ± 0.48 ^a^	2.79 ± 0.17 ^a^
Polyphenols (%)	3.52 ± 0.12 ^b^	2.63 ± 0.16 ^a^	2.77 ± 0.12 ^a^
Alginate (%)	9.54 ± 0.41 ^a^	9.60 ± 0.68 ^a^	8.84 ± 0.49 ^a^
Sulfate/fucose (molar ratio) ^1^	1.42 ± 0.09 ^b^	1.62 ± 0.10 ^c^	1.22 ± 0.04 ^a^
**Monosaccharide Compositions (Molar Ratio)**	**SC1**	**SC2**	**SC3**
Fucose	1	1	1
Mannose	0.13	0.17	0.29
Rhamnose	0.13	0.17	0.29
Glucose	ND ^3^	ND	ND
Glucuronic acid	0.38	0.67	0.71
Galactose	0.75	1.16	1.00
Xylose	0.13	0.17	0.14

**^1^** Sulfate/fucose (molar ratio) = (weight of sulfate/molecular weight of sulfate)/(weight of fucose/molecular weight of fucose); ^2^ Values are mean ± SD (n = 3); values in the same row with different letters (in ^a^, ^b^, and ^c^) are significantly different (*p* < 0.05); ^3^ ND: not detected.

## References

[1] Nordberg J., Arnér E.S. (2001). Reactive oxygen species, antioxidants, and the mammalian thioredoxin system. Free Radic. Bio. Med..

[2] Vijayabaskar P., Vaseela N., Thirumaran G. (2012). Potential antibacterial and antioxidant properties of a sulfated polysaccharide from the brown marine algae *Sargassum swartzii*. Chin. J. Nat. Med..

[3] Butterfield D.A., Drake J., Pocernich C., Castegna A. (2001). Evidence of oxidative damage in Alzheimer’s disease brain: Central role for amyloid β-peptide. Trends Mol. Med..

[4] Reed T.T. (2011). Lipid peroxidation and neurodegenerative disease. Free Radic. Biol. Med..

[5] Gupta S., Abu-Ghannam N. (2011). Bioactive potential and possible health effects of edible brown seaweeds. Trends Food Sci. Technol..

[6] Kim M.M., Rajapakse N., Kim S.K. (2009). Anti-inflammatory effect of *Ishige okamurae* ethanolic extract via inhibition of NF-κB transcription factor in RAW 264.7 cells. Phytother. Res..

[7] Pangestuti R., Kim S.K. (2011). Neuroprotective effects of marine algae. Mar. Drugs.

[8] Lee S.G., Kang H. (2015). Neuroprotective effect of *Sargassum thunbergii* (Mertens ex Roth) Kuntze in activated murine microglial cells. Trop. J. Pharm. Res..

[9] Jin W.H., Zhang W.J., Wang J., Yao J.T., Xie E., Liu D.C., Duan D., Zhang Q.B. (2014). A study of neuroprotective and antioxidant activities of heteropolysaccharides from six *Sargassum* species. Int. J. Biol. Macromol..

[10] Ale M.T., Maruyama H., Tamauchi H., Mikkelsen J., Meyer A.S. (2011). Fucoidan from *Sargassum* sp. and *Fucus vesiculosus* reduces cell viability of lung carcinoma and melanoma cells in vitro and activates natural killer cells in mice in vivo. Int. J. Biol. Macromol..

[11] Ale M.T., Mikkelsen J.D., Meyer A.S. (2011). Important determinants for fucoidan bioactivity: A critical review of structure-function relations and extraction methods for fucose-containing sulfated polysaccharides from brown seaweeds. Mar. Drugs.

[12] Gao Y.L., Dong C.H., Yin J.G., Shen J.Y., Tian J.W., Li C.M. (2012). Neuroprotective effect of fucoidan on H_2_O_2_-induced apoptosis in PC12 cells via activation of PI3K/Akt pathway. Cell. Mol. Neurobiol..

[13] Huang C.Y., Wu S.J., Yang W.N., Kuan A.W., Chen C.Y. (2016). Antioxidant activities of crude extracts of fucoidan extracted from *Sargassum glaucescens* by a compressional-puffing-hydrothermal extraction process. Food Chem..

[14] Murakami K., Yamaguchi Y., Noda K., Fujii T., Shinohara N., Ushirokawa T., Sugawa-Katayama Y., Katayama M. (2011). Seasonal variation in the chemical composition of a marine brown alga, *Sargassum horneri* (Turner) C. Agardh. J. Food Compost. Anal..

[15] Chiang P.S., Lee D.J., Whiteley C.G., Huang C.Y. (2017). Antioxidant phenolic compounds from Pinus morrisconicola using compressional-puffing pretreatment and water-ethanol extraction: Optimization of extraction parameters. J. Taiwan Inst. Chem. Eng..

[16] An Y.E., Ahn S.C., Yang D.C., Park S.J., Kim B.Y., Baik M.Y. (2011). Chemical conversion of ginsenosides in puffed red ginseng. LWT Food Sci. Technol..

[17] Varnalis A.I., Brennan J.G., MacDougall D.B. (2001). A proposed mechanism of high-temperature puffing of potato. Part I. The influence of blanching and drying conditions on the volume of puffed cubes. J. Food Eng..

[18] Moore R.E. (1977). Volatile compounds from marine algae. Acc. Chem. Res.

[19] Cho E.H., Park K.H., Kim S.Y., Oh C.S., Bang S.I., Chae H.J. (2011). Process development for deodorization of fucoidan using a combined method of solvent extraction and spray drying. KSBB J..

[20] Khalafu S.H.S., Mustapha W.A.W., Lim S.J., Maskat M.Y. (2016). The effect of deodorization on volatile compositions of fucoidan extracted from brown seaweed (*Sargassum* sp.). AIP Conf. Proc..

[21] Skriptsova A.V., Shevchenko N.M., Tarbeeva D.V., Zvyagintseva T.N. (2012). Comparative study of polysaccharides from reproductive and sterile tissues of five brown seaweeds. Mar. Biotechnol..

[22] Peranginangin R., Saepudin E. (2015). Purification and characterization of fucoidan from the brown seaweed *Sargassum binderi* Sonder. Squalen Bull. Mar. Fish. Postharvest Biotech..

[23] Wang C.Y., Wu T.C., Hsieh S.L., Tsai Y.H., Yeh C.W., Huang C.Y. (2015). Antioxidant activity and growth inhibition of human colon cancer cells by crude and purified fucoidan preparations extracted from *Sargassum cristaefolium*. J. Food Drug Anal..

[24] Zhang Z.S., Wang X.M., Zhao M.X., Yu S.C., Qi H.M. (2013). The immunological and antioxidant activities of polysaccharides extracted from *Enteromorpha linza*. Int. J. Biol. Macromol..

[25] Peng Z.F., Liu M., Fang Z.X., Wu J.L., Zhang Q.Q. (2012). Composition and cytotoxicity of a novel polysaccharide from brown alga (*Laminaria japonica*). Carbohydr. Polym..

[26] Sinha S., Astani A., Ghosh T., Schnitzler P., Ray B. (2010). Polysaccharides from *Sargassum tenerrimum*: Structural features, chemical modification and anti-viral activity. Phytochemistry.

[27] Zhou J., Hu N., Wu Y.L., Pan Y.J., Sun C.R. (2008). Preliminary studies on the chemical characterization and antioxidant properties of acidic polysaccharides from *Sargassum fusiforme*. J. Zhejiang Univ. Sci. B.

[28] Tissot B., Salpin J.Y., Martinez M., Gaigeot M.P., Daniel R. (2006). Differentiation of the fucoidan sulfated L-fucose isomers constituents by CE-ESIMS and molecular modeling. Carbohydr. Res..

[29] Li B., Lu F., Wei X.J., Zhao R.X. (2008). Fucoidan: Structure and bioactivity. Molecules.

[30] Hu M., Cui N., Bo Z.X., Xiang F.X. (2017). Structural determinant and its underlying molecular mechanism of STPC2 related to anti-angiogenic activity. Mar. Drugs.

[31] Wang J., Zhang Q.B., Zhang Z.S., Song H.F., Li P.C. (2010). Potential antioxidant and anticoagulant capacity of low molecular weight fucoidan fractions extracted from *Laminaria japonica*. Int. J. Biol. Macromol..

[32] Ananthi S., Raghavendran H.R.B., Sunil A.G., Gayathri V., Ramakrishnan G., Vasanthi H.R. (2010). In vitro antioxidant and in vivo anti-inflammatory potential of crude polysaccharide from *Turbinaria ornata* (Marine Brown Alga). Food Chem. Toxicol..

[33] Imbs T.I., Skriptsova A.V., Zvyagintseva T.N. (2015). Antioxidant activity of fucose-containing sulfated polysaccharides obtained from *Fucus evanescens* by different extraction methods. J. Appl. Phycol..

[34] Bilan M.I., Grachev A.A., Ustuzhanina N.E., Shashkov A.S., Nifantiev N.E., Usov A.I. (2002). Structure of a fucoidan from the brown seaweed *Fucus evanescens* C. Ag. Carbohyd. Res..

[35] Leong L.P., Shui G. (2002). An investigation of antioxidant capacity of fruits in Singapore markets. Food Chem..

[36] Magliaro B.C., Saldanha C.J. (2009). Clozapine protects PC-12 cells from death due to oxidative stress induced by hydrogen peroxide via a cell-type specific mechanism involving inhibition of extracellular signal-regulated kinase phosphorylation. Brain Res..

[37] Cai L., Wang H., Li Q., Qian Y.F., Yao W.B. (2008). Salidroside inhibits H_2_O_2_-induced apoptosis in PC 12 cells by preventing cytochrome c release and inactivating of caspase cascade. Acta Biochim. Biophys. Sin. (Shanghai).

[38] Guan S., Bao Y.M., Jiang B., An L.J. (2006). Protective effect of protocatechuic acid from *Alpinia oxyphylla* on hydrogen peroxide-induced oxidative PC12 cell death. Eur. J. Pharmacol..

[39] Crompton M. (1999). The mitochondrial permeability transition pore and its role in cell death. Biochem. J..

[40] Tang X.Q., Feng J.Q., Chen J., Chen P.X., Zhi J.L., Cui Y., Guo R.X., Yu H.M. (2005). Protection of oxidative preconditioning against apoptosis induced by H_2_O_2_ in PC12 cells: Mechanisms via MMP, ROS, and Bcl-2. Brain Res..

[41] Green D.R., Kroemer G. (2004). The pathophysiology of mitochondrial cell death. Science.

[42] Van Engeland M., Nieland L.J., Ramaekers F.C., Schutte B., Reutelingsperger C.P. (1998). Annexin V-affinity assay: A review on an apoptosis detection system based on phosphatidylserine exposure. Cytometry.

[43] Gibbons M.N. (1955). The determination of methylpentoses. Analyst.

[44] Filisetti-Cozzi T.M., Carpita N.C. (1991). Measurement of uronic acids without interference from neutral sugars. Anal. Biochem..

[45] Honya M., Kinoshita T., Ishikawa M., Mori H., Nisizawa K. (1993). Monthly determination of alginate, M/G ratio, mannitol, and minerals in cultivated *Laminaria japonica*. Bull. Jpn. Soc. Sci. Fish..

[46] Huang C.Y., Kuo J.M., Wu S.J., Tsai H.T. (2016). Isolation and characterization of fish scale collagen from tilapia (*Oreochromis* sp.) by a novel extrusion-hydro-extraction process. Food Chem..

